# Supplementary material for: The discovery of novel noncoding RNAs in 50 bacterial genomes
Source: Nucleic Acids Res. 2024 Apr 22;52(9):5152–65. doi: 10.1093/nar/gkae248 (PMC11109978; doi:10.1093/nar/gkae248)
Supplement: gkae248_Supplemental_Files [file gkae248_supplemental_files.zip › Narunsky 50 Genomes RNA Motifs Files.zip!/Publication/64-S_moniliformis/Figures/LRC-64-13-NC_013515_1_IGR_91_157013_157206.sto.pdf]

LRC-64-13-NC_013515_1_IGR_91_157013_157206.sto.r2r

5´- A U U C A C C U C U U C A U A U U A A C U G G U A C U A A C C A U U U A U A Y A U A U U A U A C U U U R A A A A U A U A A A A G U C A A U A U A U A R A U
